# 

**Published:** 1886-09

**Authors:** 


Page

ORIGINAL COMMUNICATIONS:

Hepatic Abscess. L. L. MacArthur ... 193
Suppurating Venereal Bubo. Scott Helm ... 201
Dipsomania and Forensic Medicine. E. C. Mann ... 207
Modification of Porro's Operation. J. Bartlett ... 211

EDITORIAL:

Professor Frank H. Hamilton. 240
The Neurological Review ... 241
Ligation of the Vertebral Arteries in Epilepsy ... 241

SOCIETY REPORTS:

CHICAGO MEDICAL SOCIETY:
*Meeting June 21.*

Intubation of the Larynx. F. E. Waxham ... 244

*Meeting July 6.*

Urinary Fistula. C. T. Parkes 260
Multilocular Ovarian Cysts. C. T. Parkes ... 261
Hydatids of the Abdomen. C. T. Parkes ... 261
Mechanical Treatment of Retroflexion of the Uterus. H. T. Byford ... 264

Page

CHICAGO MEDICAL SOCIETY:
*Meeting July 19.*

Differential Diagnosis of Scrotal Tumors. D. A. K. Steele 275
Ligation of the Vertebral Arteries in Epilepsy. J. L. Gray ... 282

*Meeting August 2.*

Diagnosis and Treatment of Hepatic Abscess. L. L. MacArthur ... 291
Subcutaneous Treatment of Buboes. Scott Helm ... 296

DR. R. C. HAMILL: *In Memoriam.* ... 298

THE CHICAGO GYNÆCOLOGICAL SOCIETY: *Meeting June 18.*

Gravid Uterus, with Adnexa. W. W. Jaggard ... 299
Ergot in Uterine Fibroids. C. T. Parkes ... 302
Treatment of Pelvic Hæmatocele. H. T. Byford ... 317

BOOK REVIEWS:

Puerperal Convalescence and the Diseases of the Puerperal Period. J. Kucher ... 321
International Medical Congress 324
Extract ... 330

